# Patient Characteristics and Utilization Patterns of Ambulatory Atrial Fibrillation Ablation in the United States

**DOI:** 10.1016/j.jacadv.2026.102741

**Published:** 2026-04-22

**Authors:** Noam Makmal, Victor Nauffal, Bruce A. Koplan

**Affiliations:** a Harvard School of Public Health, Boston, Massachusetts, USA; b Brigham and Women's Hospital, Cardiovascular Division, Boston, Massachusetts, USA

**Keywords:** ambulatory, atrial fibrillation, catheter ablation, health care utilization, racial disparities, sex disparities

## Abstract

**Background:**

Despite the growing trend toward performing catheter ablation for atrial fibrillation (AF) as an ambulatory procedure, there is limited data regarding patient characteristics and utilization patterns of ambulatory AF ablation.

**Objectives:**

The aim of the study was to evaluate national utilization patterns of ambulatory AF ablation and assess potential differences by sex, race/ethnicity, and socioeconomic status.

**Methods:**

Data were abstracted from the Nationwide Ambulatory Surgery Sample datasets (2020-2022). Ablation utilization was evaluated on a per-capita basis, with U.S. Census data used as the population denominator. Age-standardized rates (ASRs) using the 2000 U.S. standard population are reported.

**Results:**

We identified a total of 326,032 weighted ambulatory AF ablation encounters in patients aged ≥18 years. Mean age was 65.7 years, 35% were women, and 87.5% were White. In-hospital mortality was low, with a total of 13 deaths over the 3-year period. The national ASR increased by 49.5%, from 26.4 to 39.5 per 100,000 between 2020 and 2022. ASR increased from 36.4 to 54.1 per 100,000 among men (+48.4%) and from 17.1 to 26.0 per 100,000 among women (+52.3%), indicating >2-fold higher ablation rates among men. Rates were consistently higher among Whites, ranging from 2.9 to 3.8 higher ASR per 100,000 population. No single racial/ethnic minority group was disproportionately more affected than the others. Over the 3 years, 32.8% of procedures were delivered to the wealthiest income quartile, while 17.2% to the poorest quartile.

**Conclusions:**

Ambulatory AF ablation rates increased substantially across all demographic groups between 2020 and 2022. However, utilization was significantly lower in women and racial/ethnic minorities.

Atrial fibrillation (AF) is the most common sustained heart rhythm disorder and is associated with an elevated risk of stroke, heart failure, and impaired quality of life.^1^ Its incidence and prevalence are higher among individuals of European descent compared to Blacks or Asians.^1^ Catheter ablation is a well-established rhythm control strategy for AF and has demonstrated superior efficacy compared to antiarrhythmic medications.^2^^,^^3^ Advancements in the safety and effectiveness of the procedure have led to substantial growth in its utilization.^4^^,^^5^ In response to the rising demand for AF ablations, centers have increasingly transitioned to an ambulatory model, with either same-day discharge or overnight observation.^5^, ^6^, ^7^ As the use of ambulatory AF ablation continues to expand, we seek to examine utilization patterns by sex, race/ethnicity, and socioeconomic status.

## Methods

We conducted a retrospective cohort study of ambulatory AF ablation encounters using the latest releases (2020-2022) of the Nationwide Ambulatory Surgery Sample (NASS) datasets.^8^ The NASS is a national representative, calendar-year, encounter-level, administrative database of all-payer in-scope ambulatory surgeries performed in hospital-owned facilities. It is part of the Healthcare Cost and Utilization Project and was compiled by the Agency for Healthcare Research and Quality. NASS sampling design is a stratified single-stage cluster sample. Discharge weights provided by Healthcare Cost and Utilization Project were used to account for the complex survey design, ensuring unbiased national estimates when calculating sample statistics and variance.^9^

AF ablation encounters were identified using Current Procedural Terminology code 93656, and analysis was restricted to patients aged ≥18 years. The following variables were used for this analysis: age, sex, race and ethnicity, median household income for ZIP code, primary payer, comorbidities, hospital location and teaching status, hospital region, and number of hospital beds. Missing data may introduce bias and result in misleading inferences about ablation utilization patterns. Hence, missing values for age, sex, race/ethnicity, median household income for zip code, and primary payer were imputed with multiple imputation using predictive mean matching and logistic regression.^10^

Descriptive statistics are presented as mean (± standard error) for continuous variables and frequency (%) for categorical variables. Weighted proportions are presented with their 95% CIs. Due to privacy concerns, numerical results are not reported if cell size is ≤10 observations. Age, sex, and race/ethnicity-stratified AF ablation rates per 100,000 population were calculated for each calendar year (2020-2022) using the U.S. Census data of the corresponding year as the population denominator. To avoid cells with zero counts, stratification by age used 5-10 years of life intervals. Crude rates were age-standardized for the 2000 U.S. standard population using the direct standardization method. Standardized rate ratio (SRR) and standardized rate difference (SRD) were used to compare demographic groups. For sex-based comparisons, men served as the reference group. For analyses by both sexes and race/ethnicity, White men served as the reference group for male comparisons and White women served as the reference group for female comparisons. As there is no sampling error associated with the census population count, the variance of the rate is determined solely by the Poisson-distributed count. We used the delta method to derive variance estimates for the rate differences and the natural logarithm of the rate ratios, which were then used to calculate the 95% CIs. The outcomes of interest were age standardized rate (ASR), SRR, and SRD stratified by sex and race/ethnicity. Data analyses were performed using R 4.4.2/3 (R Core Team, 2018). The study was exempt from IRB approval, given that the NASS is a publicly available database with deidentified data.

## Results

**Figure 1:**
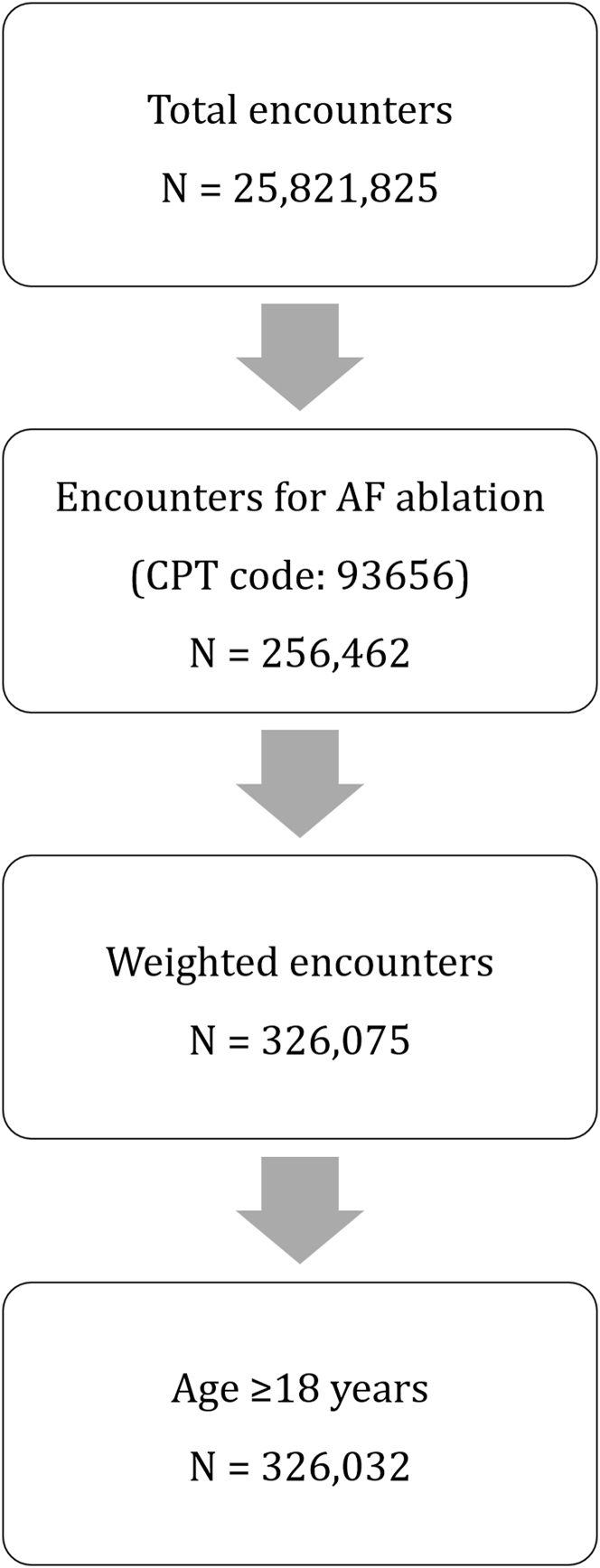
**Nationwide Ambulatory Surgery Sample Encounters Inclusion Steps (2020-2022)** Out of 25,821,825 encounters for any ambulatory surgical procedure, we identified a total of 256,462 encounters for AF ablation. This represents approximately 326,075 weighted procedures, of which 326,032 were in patients aged 18 years or older. AF = atrial fibrillation; CPT = Current Procedural Terminology.

Out of 25,821,825 encounters for any ambulatory surgical procedure that were reported between 2020 and 2022 in the NASS datasets, we identified a total of 256,462 encounters for AF ablation. This represents, after applying sampling weights to calculate the national estimates, approximately 326,075 ambulatory AF ablation procedures, of which 326,032 were in patients aged 18 years or older (Figure 1).

### AF ablations by patient characteristics

Patient characteristics stratified by year are summarized in Table 1. Imputation for missing values did not considerably alter the proportion of each subgroup (Supplemental Table 1). There was an increase of approximately 34.5% and 14.5% in procedural volume from 2020 (ASR: 26.4 [95% CI: 26.4-26.4] per 100,000) to 2021 (ASR: 35.1 [95% CI: 35.1-35.1] per 100,000) and from 2021 to 2022 (ASR: 39.5 [95% CI: 39.5-39.5] per 100,000), respectively. In-hospital mortality was low, with a total of 13 deaths over the 3-year period (<0.004%).

**Table 1:** Baseline Characteristics by Year

	2020	2021	2022
Catheter ablation, N	83,922	112,881	129,229
Age, y, mean (SE)	65.2 (0.05)	65.6 (0.05)	66.1 (0.05)
Female, n (%)	28,818 (34.34)	39,715 (35.18)	45,600 (35.29)
LAAC, n (%)	22 (0.03)	45 (0.04)	68 (0.05)
Race & ethnicity, n (%)			
White	73,958 (88.13)	98,742 (87.47)	112,568 (87.11)
Black	3,317 (3.95)	4,608 (4.08)	5,117 (3.96)
Hispanic	3,189 (3.80)	4,500 (3.99)	5,545 (4.29)
Asian/PI	1,502 (1.79)	2,191 (1.94)	2,755 (2.13)
Native American	176 (0.21)	291 (0.26)	312 (0.24)
Other	1,778 (2.12)	2,547 (2.26)	2,930 (2.27)
Median income quartile for zip code, n (%)			
Quartile 1	14,178 (16.89)	19,590 (17.35)	22,387 (17.32)
Quartile 2	20,118 (23.97)	25,783 (22.84)	30,000 (23.21)
Quartile 3	22,118 (26.36)	30,230 (26.78)	34,539 (26.73)
Quartile 4	27,507 (32.78)	37,277 (33.02)	42,302 (32.73)
Primary payer, n (%)			
Medicare	43,371 (51.7)	59,900 (53.1)	70,502 (54.6)
Medicaid	2,837 (3.38)	4,113 (3.64)	4,752 (3.68)
Private	34,855 (41.5)	45,028 (39.9)	50,133 (38.8)
Self-pay	700 (0.83)	1,065 (0.94)	959 (0.74)
No charge	36 (0.04)	39 (0.04)	36 (0.03)
Other	2,121 (2.53)	2,734 (2.42)	2,846 (2.20)
Comorbidities^a^, n (%)			
Cancer	943 (1.12)	1,203 (1.07)	1,358 (1.05)
Diabetes mellitus	15,837 (18.87)	21,476 (19.03)	23,771 (18.40)
Hypertension	55,340 (65.94)	74,402 (65.91)	82,303 (63.69)
Chronic lung disease	10,564 (12.59)	14,157 (12.54)	14,721 (11.39)
Obese	19,226 (22.91)	26,370 (23.36)	28,806 (22.29)
Peripheral vascular disease	4,564 (5.44)	6,294 (5.58)	6,842 (5.30)
Hypothyroidism	9,649 (11.50)	12,431 (11.01)	12,761 (9.88)
Hospital location and teaching status, n (%)			
Rural	1,401 (1.67)	1,965 (1.74)	2,337 (1.81)
Urban nonteaching	10,780 (12.85)	14,073 (12.47)	14,632 (11.32)
Urban teaching	71,740 (85.48)	96,843 (85.79)	112,259 (86.87)
Hospital region, n (%)			
Northeast	13,629 (16.24)	18,272 (16.19)	22,597 (17.49)
Midwest	23,526 (28.03)	33,151 (29.37)	37,664 (29.15)
South	28,167 (33.56)	37,639 (33.34)	42,100 (32.58)
West	18,599 (22.16)	23,818 (21.10)	26,867 (20.79)
Hospital bed size, n (%)			
Small (<100)	2,158 (2.57)	2,476 (2.19)	2,847 (2.20)
Medium (100-299)	17,271 (20.58)	23,375 (20.71)	27,158 (21.02)
Large (≥300)	64,492 (76.85)	87,030 (77.10)	99,223 (76.78)

LAAC = left atrial appendage closure; PI = Pacific Islander; SE = standard error.

a Comorbidities were identified using ICD-10 code. Data presented as n, n (%) or mean (SE).

Mean age increased from 65.2 (±0.05) to 66.1 (±0.05) years, and the proportion of women increased from 34.3% (95% CI: 34.1%-34.5%) to 35.3% (95% CI: 35.1%-35.5%). The vast majority of patients were White, with their proportion decreasing slightly from 88.1% (95% CI: 87.6%-88.7%) in 2020 to 87.1% in 2022 (95% CI: 86.5%-87.7%). Conversely, all racial/ethnic minority groups experienced minor increase in proportion, ranging from 0.007% to 0.49% over the same period. Evaluation of comorbidities showed that approximately two-thirds of patients had hypertension, nearly one-fifth had diabetes, and about 12% had chronic lung disease. Less than 0.06% of cases involved concomitant left atrial appendage closure.

Over the 3 years, 32.8% (95% CI: 32.0%-33.7%) of procedures were delivered to the wealthiest income quartile, while 17.2% (95% CI: 16.8%-17.7%) to the poorest quartile. Stratification by sex and race/ethnicity reveals that the proportion of men in the highest income quartile was consistently greater than that of women across all racial/ethnic groups, except for Native Americans (Supplemental Figure 1). Among Whites, Blacks, and Hispanics, the proportion of women in the lowest income quartile was consistently greater than that of men. The highest proportion of Whites and Asians/Pacific Islanders (PIs) fell within the top income quartile (30%-35% and 51%-58%, respectively), whereas the highest proportion of Blacks was observed in the lowest income quartile (35-43%).

Medicare was the expected primary payer in more than 50% of procedures, with an increase of 2.9% in proportion over the 3 years. Private insurance was the second most common primary payer, ranging from 41.5% (95% CI: 40.8%-42.2%) in 2020 to 38.8% (95% CI: 38.2%-39.4%) in 2022. The proportion of patients covered by Medicaid was considerably lower, at 3.4% (95% CI: 3.2%-3.6%) in 2020, increasing slightly to 3.7% (95% CI: 3.5%-3.8%) by 2022. While <3% of Whites had Medicaid coverage, the proportion among other racial groups was 2.5 to 4.5 times higher (Supplemental Figure 2). Most patients younger than 65 years were covered by private insurance.

Most procedures were performed in teaching urban hospitals. There was an increase of 0.7% each year in the proportion of ambulatory ablation procedures that are performed in rural hospitals.

### AF ablation rates by sex and race/ethnicity

**Figure 2:**
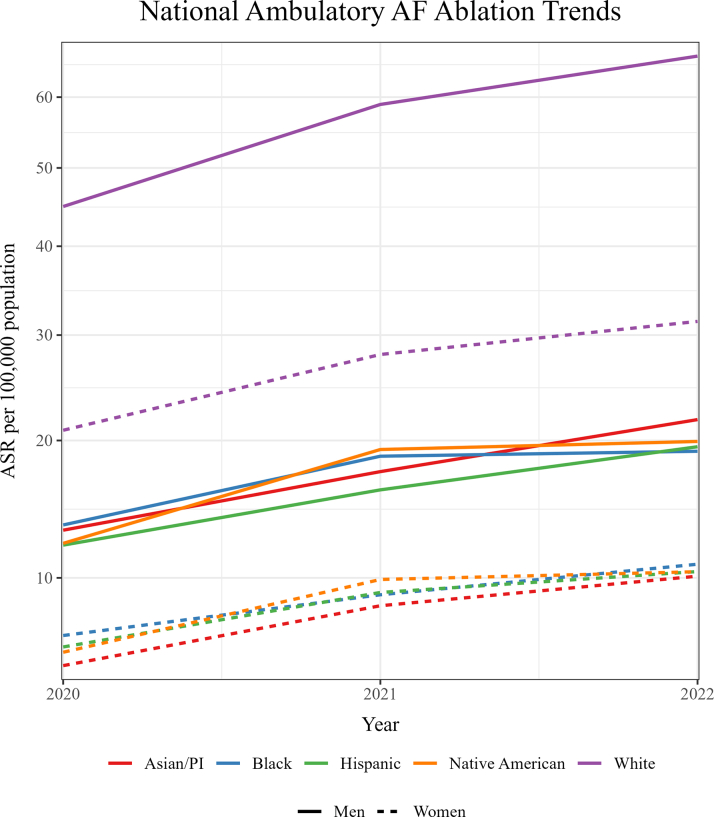
**National Ambulatory Atrial Fibrillation Ablation Rates by Sex and Race/Ethnicity** The rate for men was consistently higher than the rate for women across all racial/ethnic groups, and the rate for Whites was steadily higher than the rate for any other racial/ethnic group, across both sexes. Rates are per 100,000 population. AF = atrial fibrillation; ASR = age-standardized rate; PI = Pacific Islander.

The national ASR increased for both sexes and all racial/ethnic groups between 2020 and 2022 (Table 2). The rate for men was consistently higher than the rate for women across all racial/ethnic groups, and the rate for Whites was steadily higher than the rate for any other racial/ethnic group, across both sexes (Figure 2).

**Table 2:** Age-Standardized Ambulatory AF Ablation Rates

Females			
White	20.9 (20.9-20.9)	28.0 (28.0-28.0)	31.4 (31.4-31.4)
Black	6.8 (6.8-6.8)	9.0 (9.0-9.0)	10.8 (10.8-10.8)
Hispanic	6.3 (6.3-6.3)	9.1 (9.1-9.1)	10.4 (10.4-10.4)
Asian/PI	5.4 (5.4-5.4)	8.4 (8.4-8.4)	10.1 (10.1-10.1)
Native American	6.0 (6.0-6.0)	9.9 (9.9-9.9)	10.4 (10.4-10.4)
Males			
White	44.9 (44.9-44.9)	58.9 (58.9-58.9)	66.2 (66.2-66.2)
Black	13.4 (13.4-13.4)	18.7 (18.7-18.7)	19.1 (19.1-19.1)
Hispanic	12.1 (12.1-12.1)	16.0 (16.0-16.0)	19.5 (19.5-19.5)
Asian/PI	13.1 (13.1-13.1)	17.4 (17.4-17.4)	21.8 (21.8-21.8)
Native American	12.2 (12.2-12.2)	19.2 (19.2-19.2)	19.9 (19.9-19.9)

Ambulatory AF ablation ASR per 100,000 population by sex and race/ethnicity. Data presented as ASR (95% CI).

AF = atrial fibrillation; ASR = age-standardized rate; PI = Pacific Islander.

Between 2020 and 2021, procedural growth was greater among women, who saw a 35.3% increase in ASR (from 17.1 [95% CI: 17.1-17.1] to 23.1 [95% CI: 23.1-23.1] per 100,000), compared with a 31.5% increase among men (from 36.4 [95% CI: 36.4-36.4] to 47.9 [95% CI: 23.1-23.1] per 100,000). Growth was less pronounced in 2022, as reflected by 12.6% and 12.8% increases in ASR among women (to 26.0 [95% CI: 26.0-26.0 per 100,000] and men (to 54.1 [95% CI: 54.1-54.1] per 100,000), respectively. The men-to-women SRR narrowed from 2.13 (95% CI: 2.13-2.13) in 2020 to 2.08 (95% CI: 2.08-2.08) in 2021, and remained unchanged in 2022. On the other hand, the men-to-women SRD increased steadily from 19.4 (95% CI: 19.4-19.4) in 2020, to 24.8 (95% CI: 24.8-24.8) in 2021, to 28.1 (95% CI: 28.1-28.1) in 2022.

Native American women demonstrated the largest relative increase in ASR between 2020 and 2021, rising by 64.5%, from 6.0 (95% CI: 6.0-6.0) to 9.9 (95% CI: 9.9-9.9) per 100,000, whereas White men exhibited the smallest relative increase, rising by 31.2% from 44.9 (95% CI: 44.9-44.9) to 58.9 (95% CI: 58.9-58.9) per 100,000. Among women, ASR increased by 55.1% for Asian/PI, 45.8% for Hispanic, 34.1% for White, and 31.9% for Black patients. Among men, increases were 57.8% for Native American, 38.9% for Black, 33.3% for Asian/PI, and 32.6% for Hispanic. From 2021 to 2022, the largest relative increase in ASR was observed among Asian/PI men (+25.2%, from 17.4 [95% CI: 17.4-17.4] to 21.8 [95% CI: 21.8-21.8] per 100,000), while the smallest was noted among Black men (+2.2%, from 18.7 [95% CI: 18.7-18.7] to 19.1 [95% CI: 19.1-19.1] per 100,000). Among women, ASR increased by 20.4% for Asian/PI, 20.3% for Black, 13.6% for Hispanic, 12.3% for White, and 4.7% for Native American patients. Among men, increases were 21.6% for Hispanic, 12.4% for White, and 3.5% for Native American patients.

Rates were significantly lower among non-Whites. Nonetheless, no single racial or ethnic minority was disproportionately more affected than the others. Relative differences in ASR (Table 3), using White patients of the corresponding sex as the reference group, were most pronounced among Asian women in 2020 (SRR: 0.26; 95% CI: 0.26-0.26), Hispanic men in 2021 (SRR: 0.27; 95% CI: 0.27-0.27), and Black and Hispanic men in 2022 (SRR: 0.29; 95% CI: 0.29-0.29). SRRs improved slightly for all demographic subgroups except Black men, whose ASR was 70.1% lower than that of White men in 2020, and remained 71.2% lower in 2022. The most notable increases were observed for Asian/PI women (6% narrowing), followed by Native American women and Asian/PI men (4%), Hispanic women and Native American men (3%), Hispanic men (2%), and Black women (1%). In contrast to the narrowing in SRRs, SRDs (Table 4) (reference group: White patients of the corresponding sex), widened across all subgroups over the same period, and differences were more pronounced in men. The greatest widening was observed among Black men, whose SRD rose by 15.6 per 100,000, from 31.5 (95% CI: 31.5-31.5) per 100,000 in 2020 to 47.1 (95% CI: 47.1-47.1) per 100,000 in 2022, in favor of Whites. SRD also rose among Hispanic (by 13.9 per 100,000), Native American (by 13.6 per 100,000), and Asian/PI (by 12.5 per 100,000) men, all in favor of Whites. A similar pattern was observed among women, with increases in SRD of 6.5 per 100,000 for Black, 6.4 per 100,000 for Hispanic, 6.2 per 100,000 for Native American, and 5.9 per 100,000 for Asian/PI patients, all in favor of Whites.

**Table 3:** Standardized Rate Ratios of Ambulatory AF Ablations

	2020		2021		2022	
	Female	Male	Female	Male	Female	Male
Black	0.33 (0.33-0.33)	0.3 (0.3-0.30)	0.32 (0.32-0.32)	0.32 (0.32-0.32)	0.34 (0.34-0.34)	0.29 (0.29-0.29)
Hispanic	0.30 (0.30-0.30)	0.27 (0.27-0.27)	0.33 (0.33-0.33)	0.27 (0.27-0.27)	0.33 (0.33-0.33)	0.29 (0.29-0.29)
Asian/PI	0.26 (0.26-0.26)	0.29 (0.29-0.29)	0.30 (0.30-0.30)	0.30 (0.30-0.30)	0.32 (0.32-0.32)	0.33 (0.33-0.33)
Native American	0.29 (0.29-0.29)	0.27 (0.27-0.27)	0.35 (0.35-0.35)	0.33 (0.33-0.33)	0.33 (0.33-0.33)	0.30 (0.30-0.30)

Ambulatory AF ablation SRR per 100,000 population by sex and race/ethnicity. Data presented as SRR (95% CI). Reference group – White patients of the corresponding sex.

AF = atrial fibrillation; PI = Pacific Islander; SRR = standardized rate ratio.

**Table 4:** SRD of Ambulatory AF Ablations

	2020		2021		2022	
	Female	Male	Female	Male	Female	Male
Black	−14.1 (−14.1 to (−14.1)	−31.5 (−31.5 to −31.5)	−19.0 (−19.0 to −19.0)	−40.2 (−40.2 to −40.2)	−20.6 (−20.6 to −20.6)	−47.1 (−47.1 to −47.1)
Hispanic	−14.6 (−14.6 to −14.6)	−32.9 (−32.9 to −32.9)	−18.9 (−18.9 to −18.9)	−42.9 (−42.9 to −42.9)	−21.1 (−21.1 to −21.1)	−46.7 (−46.7 to −46.7)
Asian/PI	−15.5 (−15.5 to −15.5)	−31.9 (−31.9 to −31.8)	−19.6 (−19.6 to −19.6)	−41.5 (−41.5 to −41.5)	−21.3 (−21.3 to −21.3)	−44.4 (−44.4 to −44.4)
Native American	−14.9 (−14.9 to −14.9)	−32.7 (−32.7 to −32.7)	−18.1 (−18.1 to −18.1)	−39.7 (−39.7 to −39.7)	−21.1 (−21.1 to −21.1)	−46.3 (−46.3 to −46.3)

Ambulatory AF ablation SRD per 100,000 population by sex and race/ethnicity. Data presented as SRD (95% CI). Reference group: White patients of the corresponding sex.

SRD = standardized rate difference; other abbreviations as in Table 3.

## Discussion

In this analysis of a national representative, all-payer ambulatory surgery database we demonstrated that utilization of ambulatory AF ablation is considerably lower among women and racial/ethnic minorities (Central Illustration). Our data also reveals a 54% increase in ambulatory AF ablation volume from 2020 to 2022, with the most substantial rise occurring between 2020 and 2021, likely reflecting the easing of COVID-19-related restrictions. Prominent increases in ambulatory surgeries volume were observed for only some procedures, as defined by Clinical Classifications Software, between 2020 and 2022. Such an increase, of 27%, was noted for cardiac rhythm conversion procedures between 2020 and 2021.^11^ The analysis also suggested low in-hospital mortality, supporting the safety of ambulatory AF ablation.

**Central Illustration:**
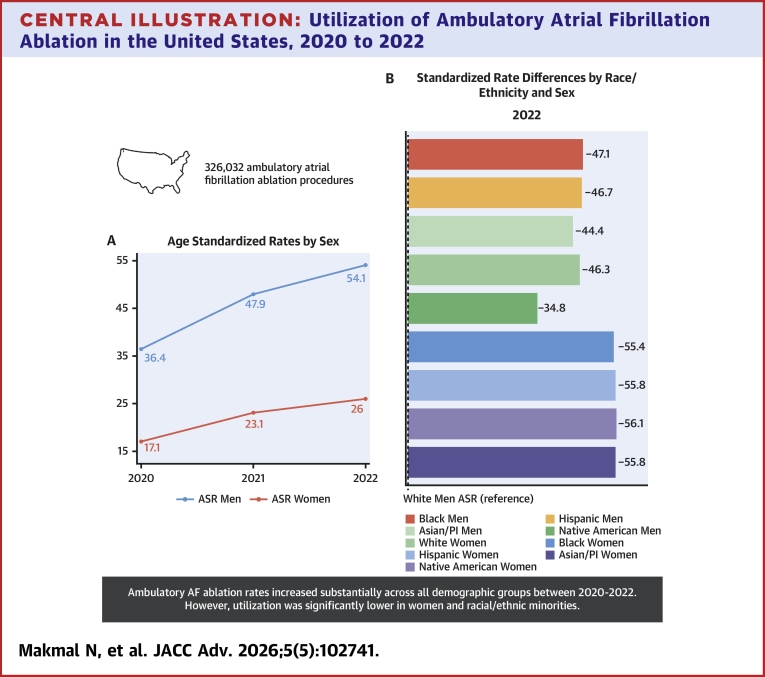
**Utilization of Ambulatory Atrial Fibrillation Ablation in the United States, 2020 to 2022** This analysis included 326,032 ambulatory AF ablation encounters of patients aged 18 years or older between 2020 and 2022. A presents sex-stratified ASRs, indicating persistently lower utilization among women and widening of the gap over time. B displays SRDs for race/ethnicity-by-sex subgroups for the year 2022, with White men as a reference group. Negative values indicating lower utilization compared to the reference group, highlighting persistent and increasing gap for women and racial/ethnic minorities. ASRs and SRDs are presented per 100,000 population. AF = atrial fibrillation; ASR = age-standardized rate; PI = Pacific Islander; SRD = standardized rate difference.

### Sex

AF prevalence is higher in men than in women. In 2021, the age-standardized prevalence of AF/flutter in the United States was 796.1 (95% uncertainty interval: 858.0-738.3) per 100,000 population in women compared to 1,329.1 (95% uncertainty interval: 1,426.7-1,246.5) per 100,000 population in men.^12^ Women with AF tend to be more symptomatic than men and report lower quality of life, yet multiple registries have reported that they are less likely to be managed with a rhythm control strategy, including ablation.^13^^,^^14^ Moreover, even when rhythm control was pursued with medications, women were more likely to convert to an interventional rate control strategy, whereas men were more likely to transition to an interventional rhythm control strategy.^15^ Notably, women often develop AF at an older age than men, approximately a decade later,^16^ which may influence patient and clinician preference away from catheter ablation, often perceived as a more aggressive therapy.

We found that AF ablation rate in men was more than twice the rate observed in women, even though the prevalence of AF is estimated to be only 67% higher. Furthermore, there was only a modest reduction in the sex-based disparity on the multiplicative scale (SRR) between 2020 and 2021, and none between 2021 and 2022. On the additive scale (SRD), sex-based differences increased steadily across the 3-year period. Although procedural growth was more prominent among women in the early COVID-19 recovery period (2020 vs 2021), further increases in ASR were similar for men and women (2021 vs 2022). Prior studies reported conflicting results. Kummer et al^17^ (HR: 0.83 [95% CI: 0.80-0.86]), Bhave et al^18^ (HR: 0.65 [95% CI: 0.63-0.68]), and Patel et al^19^ (OR: 0.83 [95% CI: 0.79-0.87]) reported significant lower likelihood of AF ablation among women. Interestingly, Yarrarapu et al^20^ observed an opposite trend (OR_men_: 0.88 [95% CI: 0.87-0.90]), which was not demonstrated in a later report by Johal et al^21^ (OR_women_: 0.73 [95% CI: 0.70-0.76]). Among rhythm control strategies, Gehi et al^22^ found that men were more likely to undergo AF ablation (OR: 1.15 [95% CI: 1.11-1.20]), whereas Eberly et al^23^ and Jackson et al^24^ did not find significant differences between the 2 groups.

### Race and ethnicity

AF incidence and prevalence are higher in Whites than in Blacks, Hispanics, or Asians, despite having lower burden of AF risk factors. It has been postulated that those differences stem from unmeasured confounders, such as barriers in access to health care among minorities leading to underdetection of AF, enrollment bias, or differential mortality.^1^^,^^25^ Increasing evidence suggests genetic component, including racial variations in left atrial size, genetic polymorphisms, epigenetic modifications, and atrial membrane stability.^26^, ^27^, ^28^ This is further supported by a large, longitudinal study demonstrating higher risk of incidental AF among Native Americans compared to the above-mentioned racial/ethnic groups.^29^

Relative increases in ASR were most pronounced in the early COVID-19 recovery period (2020 vs 2021) for all racial/ethnic groups. Growth patterns differed between 2020 to 2021 and 2021 to 2022, but tended to be greatest in groups with lower baseline rates. We observed marked differences in AF ablation rates across racial/ethnic groups. Whites consistently had the highest utilization. SRRs range from 2.9 to 3.8 per 100,000 population in favor of Whites, with only minor improvement for minorities over the 3-year period. SRR increased for most ethnic/racial groups between 2020 and 2021, whereas changes were more heterogenous between 2021 and 2022. SRDs consistently widened over time in favor of Whites, and differences were more pronounced among men. No single racial or ethnic minority groups stood out as being disproportionately more affected than the others. Although genetic factors may contribute to variations in AF incidence and prevalence, and consequently to differences in ablation rates, the magnitude of the observed differences is unlikely to be explained by biological factors alone. It is therefore plausible that additional factors, such as disparities in access to care, might play a role. Our findings are in line with previous studies. In analyses of all-payers data,^17^^,^^19^, ^20^, ^21^^,^^30^ which primarily reflect ablations performed in inpatient settings, Blacks and Hispanics were less likely to undergo AF ablation compared to Whites. These differences were more pronounced for Black patients, who were 20 to 51% less likely to receive ablation therapy compared with White patients, whereas Hispanics were 17 to 40% less likely. Results from other sources vary. Bhave et al^18^ reported that among Medicare beneficiaries, Hispanics (HR: 0.70 [95% CI: 0.63-0.79]), but not Blacks, were less likely to undergo ablation compared to Whites. In analyses restricted to rhythm control strategies, with Whites as the reference group, Eberly et al^23^ observed lower odds of ablation among Hispanics (OR: 0.73 [95% CI: 0.60-0.89]), but not Blacks. On the other hand, Jackson et al^24^ found that Black patients (OR: 0.80 [95% CI: 0.68-0.94]), but not Hispanic patients, were less likely to undergo ablation within 1-year of AF diagnosis. Kummer et al,^17^ Gu et al,^31^ Yarrarapu et al,^20^ and Johal et al^21^ found that Asians were 18 to 42% less likely to be treated with catheter ablation compared to Whites. Eberly et al^23^ and Jackson et al^24^ did not observe significant differences in utilization of catheter ablation among Asian and White patients receiving rhythm control therapies. Existing literature regarding AF management in Native Americans is scant. While Jackson et al^24^ (OR: 1.39 [95% CI: 1.25-1.55]) reported higher odds of catheter ablation in Native Americans than in Whites, Johal et al^21^ (OR: 0.81 [95% CI: 0.59-1.11]) did not find significant difference.

### Socioeconomic status

Low-income patients are less likely to receive advanced cardiovascular services,^32^^,^^33^ and access to medical care is strongly tied to individual's insurance type. Prior studies have highlighted the association between lower utilization of innovative technologies in health care and disadvantageous insurance status.^34^, ^35^, ^36^, ^37^

We observed that majority of patients undergoing ambulatory AF ablation were from the highest household income quartile, with proportions decreasing progressively across lower income groups. While unadjusted, this finding is consistent with most available literature.^17^^,^^20^^,^^21^^,^^23^

Our analysis indicate that Medicare was the dominant expected payer, reflecting the older age of the cohort, while Medicaid accounted for a small share overall. Among patients younger than 65, private insurance was the most common expected payer. The proportion of Medicaid beneficiaries was considerably higher among minorities compared to Whites, which highlights potential underlying socioeconomic differences by race. Previous studies present inconsistent results. Kummer et al^17^ and Patel et al^19^ found that in inpatient settings, patients with private insurance were most likely to be treated with AF ablation. Although Yarrarapu et al^20^ analyzed data from overlapping years, they found no differences in utilization of AF ablation among hospitalized patients with private insurance, Medicare, and Medicaid. In a more recent analysis, Johal et al^21^ observed no differences between the private insurance and Medicare groups, but reported lower odds of AF ablation among Medicaid beneficiaries (OR: 0.66 [95% CI: 0.60-0.72]) compared to Medicare beneficiaries.

### Study Limitations

Several limitations should be acknowledged. First, ablation utilization was assessed per population rather than standardized to AF prevalence of ablation-eligible population. In addition, data on comorbidities, determinants of ablation candidacy, and medication use were largely unavailable, limiting our ability to fully characterize the study population and, in turn, constraining interpretation of the findings. Hence, the observed disparities may reflect a combination of AF prevalence, referral patterns, access to care, and patient selection. Second, our cohort includes only patients who experienced the outcome of interest (ie ambulatory AF ablation) and the use of comparative group, to strengthen causal inference, was not feasible. Lastly, the NASS does not capture patients who were admitted to the same hospital, where the ambulatory procedure was performed, thereby precluding assessment of serious procedure-related complications occurring after the index procedure in the admitted patients.

## Conclusions

In an analysis of all-payer, national representative dataset, we observed a substantial increase in ambulatory AF ablation procedures across all sex and racial/ethnic groups. Despite overall procedural growth, persistent differences in utilization were evident, with lower ablation rates among women and racial/ethnic minorities. Relative differences showed minor improvement, whereas absolute differences widened. Although genetic factors related to the risk of AF development may contribute to the sex- and race/ethnicity-based variation in AF ambulatory ablation rates, the observed differences are unlikely to be explained by biological factors alone. The findings of our study highlight the need for further research to better understand the underlying reasons for these differences and to inform strategies that promote access to advanced rhythm control therapies.

Perspectives **COMPETENCY IN SYSTEMS-BASED PRACTICE:** Identify and address financial, cultural, and social barriers to evaluation and management recommendations and adherence.

**COMPETENCY IN PATHOPHYSIOLOGICAL BASIS OF CARDIAC ARRHYTHMIAS/BASIC ELECTROPHYSIOLOGY:** Know the epidemiology of arrhythmias.

**TRANSLATIONAL OUTLOOK 1:** Despite the advantage of large dataset, limited clinical details in the NASS hinders the ability to fully assess patient risk factors and outcomes. Future research should not rely solely on administrative data.

**TRANSLATIONAL OUTLOOK 2:** Differences in utilization by sex and race/ethnicity remained unexplained. Further studies should investigate underlying access-related factors to inform interventions that promote equity.

## Funding support and author disclosures

Dr Koplan has served as a consultant for 10.13039/100006775GE Healthcare and 10.13039/100008497Boston Scientific. Dr Nauffal has served as a consultant for 10.13039/100000046Abbott and is supported by a grant from 10.13039/501100004191Novo Nordisk focused on identifying therapeutic targets for cardiac fibrosis. Dr Makmal has reported that they have no relationships relevant to the contents of this paper to disclose.
