# Supplementary material for: Patient Characteristics and Utilization Patterns of Ambulatory Atrial Fibrillation Ablation in the United States
Source: JACC Adv. 2026 Apr 22;5(5):102741. doi: 10.1016/j.jacadv.2026.102741 (PMC13126487; doi:10.1016/j.jacadv.2026.102741)
Supplement: Supplemental Material [file mmc1.docx]

Supplemental Table 1: Pre-imputation data

|  | **2020** | **2021** | **2022** |
| --- | --- | --- | --- |
| Age, years - mean (SD) | 65.2 (0.05) | 65.6 (0.05) | 66.1 (0.05) |
| Female - n (%) | 28,817 (34.34) | 39,699 (35.18) | 45,597 (35.29) |
| **Race & Ethnicity - n (%)** | | | |
| White | 72,592 (88.38) | 96,448 (87.69) | 110,058 (87.18) |
| Black | 3,196 (3.89) | 4,351 (3.96) | 4,956 (3.93) |
| Hispanic | 3,009 (3.66) | 4,312 (3.92) | 5,359 (4.24) |
| Asian / PI | 1,442 (1.76) | 2,139 (1.94) | 2,694 (2.13) |
| Native American | 172 (0.21) | 287 (0.26) | 308 (0.24) |
| Other | 1,723 (2.10) | 2,445 (2.22) | 2,870 (2.27) |
| **Median Income Quartile for Zip Code - n (%)** | | | |
| Quartile 1 | 13,982 (16.86) | 19,312 (17.31) | 22,145 (17.30) |
| Quartile 2 | 19,847 (23.93) | 25,452 (22.81) | 29,701 (23.20) |
| Quartile 3 | 21,837 (26.33) | 29,877 (26.77) | 34,219 (26.73) |
| Quartile 4 | 27,285 (32.89) | 36,950 (33.11) | 41,969 (32.78) |
| **Primary Payer - n (%)** | | | |
| Medicare | 43,346 (51.69) | 59,856 (53.07) | 70,471 (54.57) |
| Medicaid | 2,832 (3.38) | 4,109 (3.64) | 4,744 (3.67) |
| Private | 34,825 (41.53) | 45,002 (39.90) | 50,110 (38.80) |
| Self-pay | 700 (0.83) | 1,061 (0.94) | 953 (0.74) |
| No charge | 33 (0.04) | 38 (0.03) | 32 (0.02) |
| Other | 2,120 (2.53) | 2,725 (2.42) | 2,838 (2.20) |

Data presented as n (%) or mean (SD).

PI, Pacific Islander; SD, Standard Deviation.

Supplemental Figure 1: Ambulatory AF ablation by household income quartile, sex, and race/ethnicity.


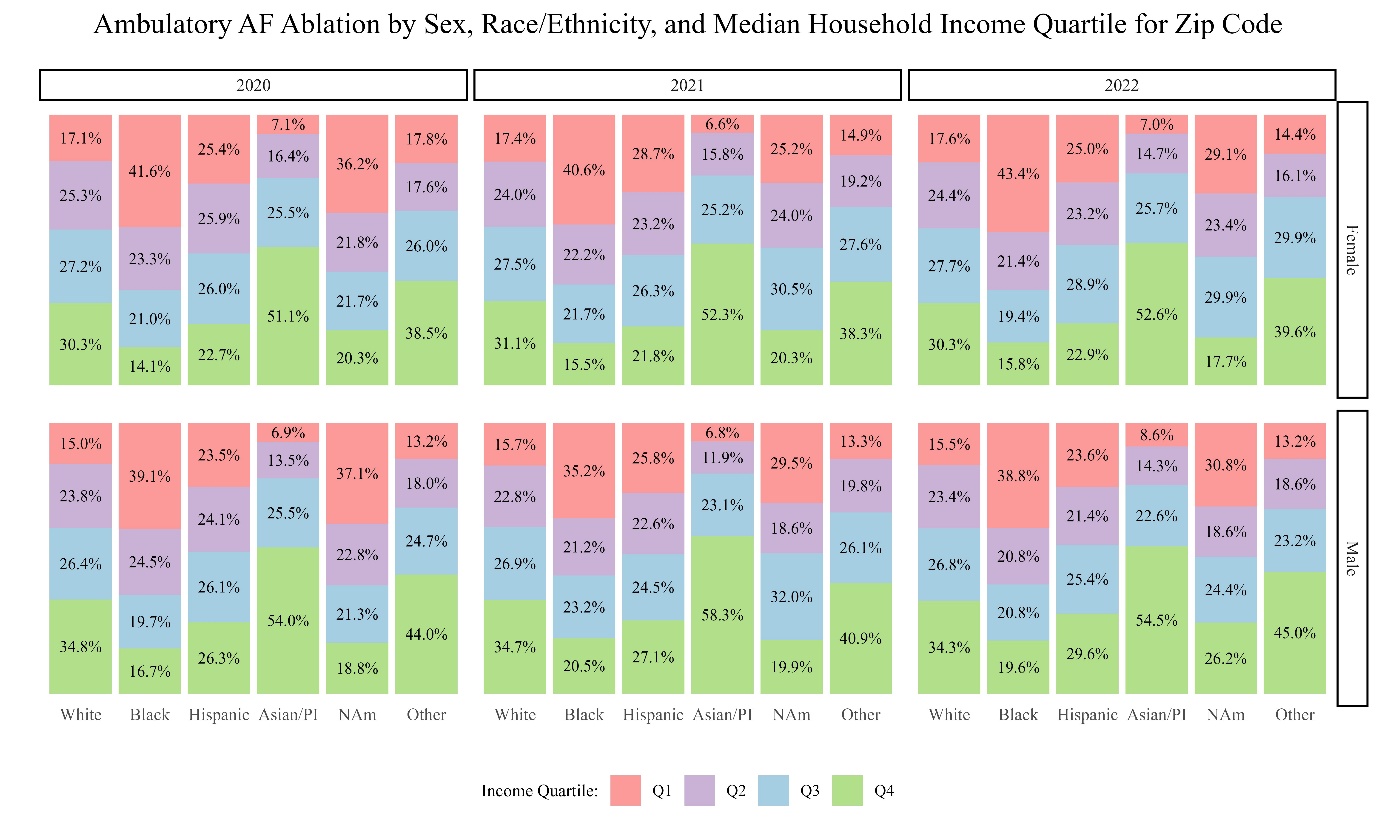


Ambulatory AF ablation by median household income quartile for ZIP code, sex, and race/ethnicity. Among Whites, Blacks, Hispanics, and Asians/PI the proportion of males in the top income quartile consistently exceeded that of females, while the percentage of females in the lowest income quartile was higher than that of males. Most Whites and Asians/PI fell within the top income quartile, whereas the largest proportion of Blacks was observed in the lowest quartile.

AF, Atrial Fibrillation; NAm, Native American; PI, Pacific Islander.

Supplemental Figure 2: Ambulatory AF ablation by primary payer and race/ethnicity


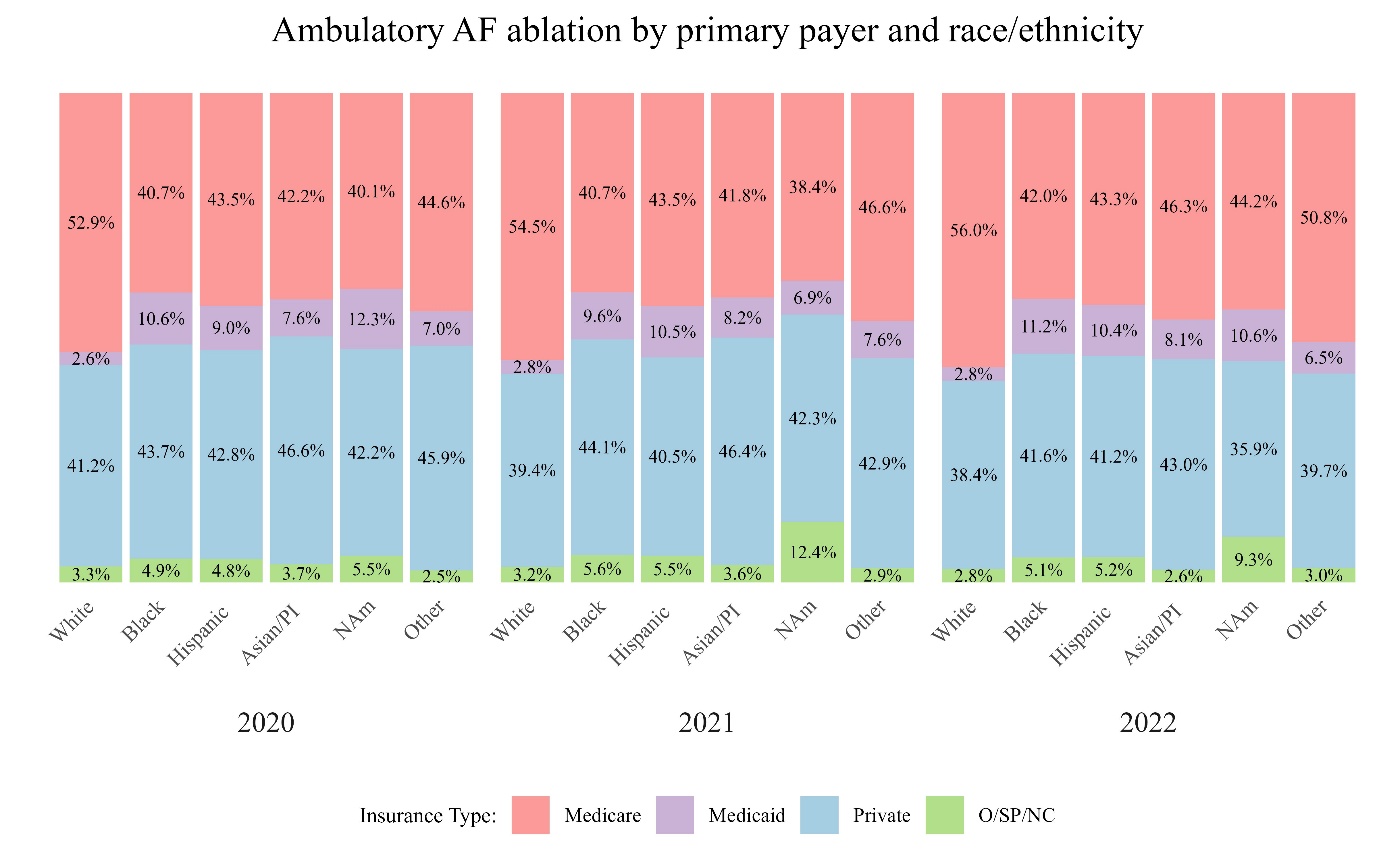


Most patients were covered by Medicare or private insurance. While the proportion of Whites covered by Medicaid did not exceed 3%, the proportions of other Race/Ethnic groups were 2.5 to 4.5 times higher. ‘Self-Pay’, ‘No Charge’ and ‘Other’ categories were combined into a single group due to patient privacy considerations.

AF, Atrial Fibrillation; NAm, Native American; NC, No Charge; O, Other; PI, Pacific Islander; SP, Self-Pay.
